# Interaction between *GSTP1* Val Allele and *H. pylori* Infection, Smoking and Alcohol Consumption and Risk of Gastric Cancer among the Chinese Population

**DOI:** 10.1371/journal.pone.0047178

**Published:** 2012-10-15

**Authors:** Ye Zhang, Li-Ping Sun, Cheng-Zhong Xing, Qian Xu, Cai-Yun He, Ping Li, Yue-Hua Gong, Yun-Peng Liu, Yuan Yuan

**Affiliations:** 1 Tumor Etiology and Screening Department of Cancer Institute and General Surgery, The First Affiliated Hospital of China Medical University, and Key Laboratory of Cancer Control in Liaoning Province, Shenyang, China; 2 Department of Medical Oncology, The First Affiliated Hospital of China Medical University, Shenyang, China; The University of Hong Kong, China

## Abstract

Glutathione S-transferase P1 (GSTP1) is a critical enzyme in the phase II detoxification pathway. One of the common functional polymorphisms of *GSTP1* is A→G at nucleotide 313, which results in an amino acid substitution (Ile105Val) at the substrate binding site and reduced catalytic activity. We evaluated the interaction between *GSTP1* Val allele and *Helicobacter pylori* infection, smoking and alcohol consumption, increasing the risk of gastric cancer among the Chinese population. Information on potential gastric cancer risk factors and blood specimens were collected from 618 incident gastric cancer cases and 1,830 non-cancer controls between March 2002 and December 2011 in Liaoning Province, China. *GSTP1* Ile105Val was genotyped by matrix-assisted laser desorption/ionization time-of-flight mass spectrometry and polymerase chain reaction-restriction fragment length polymorphism. Serum levels of anti-*H. pylori* IgG were measured by ELISA. Odds ratio (OR) and 95% confidence interval (CI) were calculated using multivariate logistic regression, adjusted by sex and age. The risk of gastric cancer was significantly elevated in patients with the *GSTP1* Val/Val genotype (adjusted OR = 3.324; 95% CI = 1.790–6.172). An elevated risk of gastric cancer was observed in patients with *H. pylori* infection, smoking, or alcohol consumption, and together with the *GSTP1* Ile/Val +Val/Val genotype (OR = 3.696; 95% CI = 2.475–5.521; OR = 1.638; 95% CI = 1.044–2.571; OR = 1.641; 95% CI = 0.983–2.739, respectively) (*p*<0.05). The *GSTP1* Val allele shows an interaction with smoking, alcohol consumption, and especially *H. pylori* infection for increasing the risk of gastric cancer. These findings could demonstrate new pathophysiological pathways for the development of gastric cancer.

## Introduction

Gastric cancer is one of the most frequently occurring cancers globally. A total of 989,600 new cases and 738,000 deaths are estimated to have occurred in 2008, accounting for 8% of the total number of cancer cases and 10% of cancer-related deaths [1]. The geographical distribution of gastric cancer exhibits wide international variation and >70% of new cases and deaths occur in developing countries. Investigations into the pathogenesis of gastric cancer have resulted in increasing evidence to suggest that interaction between various inherited cancer susceptibility genes could affect an individual’s risk of developing gastric cancer [2]. These genes are also known as risk-modifier genes, particularly those whose allelic polymorphisms are responsible for impaired metabolism of environmental carcinogens and/or repair of oxidative-stress-induced DNA damage.

Since the first description by Krontiris in 1985 that the polymorphisms of the *RAS* gene can be used to assess the risk of oncogenesis [3], more studies have begun to demonstrate associations between the polymorphisms and gastric cancer susceptibility, including oncogenes [4], antioncogenes [5], [6] and immunomodifier genes [7], [8]. It has also been suggested that genetic susceptibility genes, especially genes for metabolic enzymes, may confer a risk for the development of gastric cancer [9]–[11]. Glutathione S-transferases (GST_S_) consist of a superfamily of dimeric phase II metabolic enzymes [12]. Several polymorphisms in GST genes result in reduced or no activity of the enzymes. Specifically, *GSTM1* and *GSTT1* genes are polymorphically deleted [13], [14]. The polymorphism *GSTP1*, c.313A>G determines the amino acid substitution Ile(105)Val, which results in reduced activity of the Val-containing enzyme [15], [16]. The polymorphisms in *GSTM1* and *GSTT1* have been investigated in cancer for a long time; however, only recently have some studies investigated the association of various cancers with *GSTP1* variants. Some studies have observed a different relation between the *GSTP1* polymorphism and an increased risk for oral [17], breast [15], [18], colorectal [19], prostate [20] and lung [21], [22] cancers. Regarding gastric cancer, only a few studies were conducted to investigate its association with *GSTP1* variants [23].

It has been increasingly accepted that the etiology of most common tumors involves not only genetic and environmental causes, but also interactions between the two. The finding that the strength of association between lifestyle and cancer occurrence is influenced by genotypes (gene–environment interaction) has opened the door to genotype applications for tumor prevention.

In this case–control molecular epidemiological study in a Chinese population we determined whether this *GSTP1* polymorphism influenced susceptibility to gastric cancer, as well as the interaction between the polymorphism and environmental factors (*Helicobacter pylori* infection, smoking, and alcohol consumption) involved in gastric carcinogenesis.

## Materials and Methods

### Study Population

A total of 2,448 patients with different gastric diseases were enrolled in this study, including gastric cancer (n = 618), atrophic gastritis (n = 683), and superficial gastritis as a control (n = 1,147). All patients were consecutively recruited from March 2002 to December 2011 in Liaoning Province, Northeast China, and were surveyed about their history of any illness. Before commencement of the study, approval was obtained from the Human Ethics Review Committee of China Medical University. Written informed consents were obtained from participants in accordance with the Declaration of Helsinki and its later revision.

### Data Collection and Laboratory Protocol

Cases and controls were interviewed face-to-face by trained interviewers using the same questionnaire. The diagnosis of gastric disease was established by upper gastrointestinal endoscopic examination and confirmed by histopathology. Histopathological findings were assessed according to the visual analog scale of the updated Sydney System [24]. Meanwhile, 5 ml of blood was collected from each patient to isolate DNA and measure serum *H. pylori* IgG. The blood samples were allowed to clot for 30–40 min at room temperature, and then centrifuged at 3,000 rpm for 10 min. The clots and sera were stored immediately at –20°C, and then moved into a freezer at –70°C within 3 days of collection.

All genomic DNA was extracted using the standard phenol–chloroform extraction procedure. In 915 cases, *GSTP1* Ile105Val single nucleotide polymorphism was genotyped by polymerase chain reaction-restriction fragment length polymorphism according to Watson et al [25], with a slight modification. In 1,595 cases, *GSTP1* was genotyped using a matrix-assisted laser desorption/ionization time-of-flight (MALDI-TOF) mass spectrometry platform (Sequenom, San Diego, CA, USA), according to the manufacturer’s instructions. Duplicate samples (62 cases, 2.5% of the total) were included for the evaluation of genotyping quality and the concordance rate was 100%. Genotyping was successfully performed among 2,448 cases. No deviation from the Hardy–Weinbery equilibrium was observed (*p* = 0.946, χ^2^ = 0.005 in control group). Finally, there were 550 gastric cancer cases and 550 superficial gastritis controls, and 683 atrophic gastritis cases and 683 superficial gastritis controls included for analysis; controls were frequency-matched with cases by sex and age±5 years.

Serum *H. pylori* IgG antibody concentrations were each measured with ELISA (*Helicobacter pylori* IgG Kit; Biohit, Helsinki, Finland). An individual was considered positive if the IgG titer was above 34 EIU.

### Statistical Analysis

All statistical analysis was performed using the SPSS 17.0 statistical software (Chicago, IL, USA). Student’s *t*-test and Pearson’s χ^2^ test were used to test for differences between groups. The association between the *GSTP1* polymorphism and risk of gastric cancer and atrophic gastritis was estimated by odds ratios (ORs) and 95% confidence intervals (CIs) adjusted by sex and age, together with the test for an interaction between the *GSTP1* polymorphism and *H. pylori* infection. *P*≤0.05 was considered statistically significant.

## Results

### Characteristics of the Study Population

The frequency distributions of demographic and other selected characteristics of the participants are shown in Table 1. There was no significant difference in the distributions of age, sex, smoking status, and alcohol consumption between the superficial gastritis controls and atrophic gastritis cases. The *H. pylori*-positive rates of the superficial gastritis control and atrophic gastritis groups, the superficial gastritis control and gastric cancer groups were 25.8% (176 of 683) *vs* 60.8% (415 of 683), and 24.4% (134 of 550) *vs* 49.3% (271 of 550), respectively, and a significant difference was observed in the atrophic gastritis group compared to the superficial gastritis controls, and the gastric cancer group compared to the superficial gastritis controls. The smoking and alcohol consumption rates were of significantly different between the superficial gastritis controls and gastric cancer cases.

**Table 1:** Distribution of select patient characteristics.

Variable	superficial gastritis *vs.* atrophic gastritis			superficial gastritis *vs.* gastric cancer		
	superficialgastritis (n = 683)	atrophicgastritis (n = 683)	*P* value^a^	superficialgastritis (n = 550)	gastriccancer (n = 550)	*P* value^a^
**Age (mean±SD),** **years**	55.16±9.26	55.05±9.21	0.824	56.63±9.77	57.22±10.17	0.328
≤60	494	496	0.904	364	344	0.208
>60	189	187		186	206	
**Sex**						
Male	393	393	1.000	373	373	1.000
Female	290	290		177	177	
***H. pylori***						
Positive	176	415	**0.000** *	134	271	**0.000** *
Negative	507	268		416	279	
**Smoking status**						
Smoker	185	181	0.212	171	141	**0.002** *
Non-smoker	320	368		213	106	
Missing	178	134		166	303	
**Alcohol use**						
Yes	122	129	0.687	111	84	**0.002** *
No	372	417		264	115	
Missing	189	137		175	351	

a *P* value of the comparison with a two-sided χ^2^ test.

* Indicates statistical significance at *P*<0.05.

### Association between *GSTP1* Genotype and Risk of Gastric Cancer

To examine whether the risks of gastric cancer and its precancerous conditions were related to *GSTP1* genotype, we analyzed the association between *GSTP1* genotype and the risk of atrophic gastritis and gastric cancer. Compared with the patients with Ile/Ile genotype, those with Ile/Val or Val/Val genotype did not harbor a significantly higher risk of atrophic gastritis. However, in the gastric cancer group, using Ile/Ile genotype as a reference (OR = 1.00), the OR for Val/Val genotype was 3.324 (95% CI = 1.790–6.172), which showed a statistically significant increase in gastric cancer risk associated with Val/Val genotype (Table 2).

**Table 2:** Genotype distribution of *GSTP1* among atrophic gastritis and gastric cancer cases and superficial gastritis controls and association with gastric cancer risk.

	superficial gastritis *vs.* atrophic gastritis				superficial gastritis *vs* gastric cancer			
	superficialgastritis	atrophicgastritis	AdjustedOR (95% CI)	*P*	superficial gastritis	gastriccancer	AdjustedOR(95% CI)	*P*
**Ile/Ile**	421	430	1.000		343	331	1.000	
**Ile/Val**	247	230	1.096(0.876–1.372)	0.422	193	174	0.929(0.720–1.199)	0.571
**Val/Val**	15	23	0.666(0.343–1.294)	0.230	14	45	**3.324(1.790–6.172)**	**0.000**

Statistically significant results (*P*<0.05) are highlighted in bold.

a ORs were calculated by logistic regression, and adjusted for age and sex.

### Stratified Analysis

We further evaluated the association between the genotypes of *GSTP1* and risk of gastric cancer and its precancerous conditions by subgroups of age, sex, *H. pylori* infection, smoking status, and alcohol consumption. No significant differences were found in the atrophic gastritis group, except in the subgroup aged >60 years (Table 3). In general, an increased atrophic gastritis risk associated with Ile/Val genotypes was more evident in subgroups aged >60 years (adjusted OR = 1.824, 95% CI = 1.185–2.809). Moreover, patients with *GSTP1* Val/Val genotypes were associated with risk of gastric cancer in almost all subgroups except for non-smoking or non-alcohol consumption subgroups (adjusted OR and 95% CI, see Table 3).

**Table 3:** Stratification analysis of association between *GSTP1* genotype and gastric cancer risk.

Variables	Genotype	Superficialgastritis *vs.*atrophicgastritis	OR(95% CI)	*P value*	Superficialgastritis *vs.*gastriccancer	OR (95% CI)	*P* value
**Age**/year							
≤60	Ile/Ile	317/302	1.000		238/212	1.000	
	Ile/Val	167/176	0.904(0.694–1.177)	0.454	117/104	1.001(0.725–1.382)	0.997
	Val/Val	10/18	0.529(0.24–1.165)	0.114	9/28	3.482(1.606–7.547)	0.002
>60	Ile/Ile	104/128	1.000		105/119	1.000	
	Ile/Val	80/54	1.824(1.185–2.809)	0.006	76/70	0.815(0.537–1.238)	0.337
	Val/Val	5/5	1.232(0.347–4.373)	0.746	5/17	2.981(1.063–8.364)	0.038
**Sex**							
Male	Ile/Ile	238/244	1.000		226/228	1.000	
	Ile/Val	146/136	0.909(0.677–1.22)	0.524	138/115	0.821(0.603–1.119)	0.212
	Val/Val	9/13	1.408(0.591–3.356)	0.44	9/30	3.297(1.53–7.104)	0.002
Female	Ile/Ile	183/186	1.000		117/103		
	Ile/Val	101/94	0.921(0.65–1.305)	0.645	55/59	1.213(0.771–1.909)	0.404
	Val/Val	6/10	10.641(0.584–4.609)	0.347	5/15	3.402(1.195–9.687)	0.022
***H. pylori***							
Negative	Ile/Ile	311/175	1.000		253/168	1.000	
	Ile/Val	186/84	0.804(0.585–1.106)	0.180	153/92	0.887(0.64–1.228)	0.470
	Val/Val	10/9	1.600(0.638–4.014)	0.317	10/19	2.788(1.263–6.153)	0.011
Positive	Ile/Ile	110/255	1.000		90/163	1.000	
	Ile/Val	61/146	1.036(0.713–1.506)	0.852	40/82	1.129(0.714–1.784)	0.603
	Val/Val	5/14	1.210(0.425–3.443)	0.721	4/26	3.538(1.196–10.47)	0.022
**Smoking**							
Neative	Ile/Ile	197/233	1.000		136/69	1.000	
	Ile/Val	117/122	0.884(0.644–1.214)	0.446	72/32	0.879(0.525–1.473)	0.625
	Val/Val	6/13	1.828(0.682–4.898)	0.231	5/5	1.841(0.505–6.709)	0.355
Positive	Ile/Ile	107/109	1.000		100/82	1.000	
	Ile/Val	74/70	0.921(0.602–1.41)	0.705	67/47	0.87(0.539–1.407)	0.571
	Val/Val	4/2	0.493(0.088–2.77)	0.422	4/12	3.638(1.122–11.79)	0.031
**Alcohols**							
Negative	Ile/Ile	231/263	1.000		170/74	1.000	
	Ile/Val	132/142	0.948(0.705–1.275)	0.725	86/32	0.836(0.508–1.376)	0.482
	Val/Val	9/12	1.167(0.483–2.823)	0.731	8/9	2.635(0.965–7.197)	0.059
Positive	Ile/Ile	69/77	1.000		62/49	1.000	
	Ile/Val	52/49	0.847(0.509–1.41)	0.523	48/28	0.744(0.404–1.371)	0.343
	Val/Val	1/3	2.657(0.27–26.175)	0.402	1/7	9.88(1.173–83.22)	0.035

### Characteristics of Patients with/without *H. pylori* Infection, Smoking, and Alcohol Consumption in Relation to *GSTP1* Polymorphisms

A variant of *GSTP1* has a lower efficiency for most of the environmental carcinogens (e.g. *H. pylori* infection, smoking, and alcohol consumption) that may cause some individuals’ susceptibility to gastric cancer and its precancerous conditions [26]–[28]. Therefore, we looked for interaction between *GSTP1* genotype and *H. pylori* IgG, smoking, or alcohol consumption in gastric cancer and its precancerous conditions. Using Ile/Ile genotype and *H. pylori* IgG(–) as a reference, the OR for (Ile/Val +Val/Val) genotype and *H. pylori* IgG(+) was 4.308 (95% CI = 3.062–6.061) in atrophic gastritis subgroups, and the OR for (Ile/Val +Val/Val) genotype and *H. pylori* IgG(+) was 3.696 (95% CI = 2.475–5.521) in gastric cancer subgroups. Using Ile/Ile genotype and non-smoking as a reference, the OR for (Ile/Val +Val/Val) genotype and smoking was 0.782 (95% CI = 0.538–1.136) in atrophic gastritis subgroups, and the OR for (Ile/Val +Val/Val) genotype and smoking was 1.638 (95% CI = 1.044–2.571) in gastric cancer subgroups. Using Ile/Ile genotype and non-alcohol consumption as a reference, the OR for (Ile/Val +Val/Val) genotype and alcohol consumption was 0.862 (95% CI = 0.565–1.313) in atrophic gastritis subgroups, and the OR for (Ile/Val +Val/Val) genotype and alcohol consumption was 1.641 (95% CI = 0.983–2.739) in gastric cancer subgroups. Association of the *GSTP1* Val/Val genotype with *H. pylori* IgG(+), smoking, or alcohol consumption could significantly increase atrophic gastritis and gastric cancer risk (Tables 4 and 5).

**Table 4:** Interaction between *GSTP1* Ile/Val polymorphism and *H. pylori* infection, smoking, and alcohol consumption in atrophic gastritis.

			superficial gastritis *vs.* atrophic gastritis			
			Ile/Ile	Ile/Val	Val/Val	Ile/Val + Val/Val
***H. pylori***	(–)	superficial gastritis/atrophic gastritis	311/175	186/84	10/9	196/93
		OR (95% CI)	1.000	0.803(0.584–1.102)	1.599(0.638–4.011)	0.843(0.619–1.148)
	(+)	superficial gastritis/atrophic gastritis	110/255	61/146	5/14	66/160
		OR (95% CI)	4.12(3.082–5.508)	4.253(2.993–6.045)	4.976(1.763–14.047)	4.308(3.062–6.061)
				***P*** ** = 0.000**	***P*** ** = 0.000**	***P*** ** = 0.000**
**Smoking**	(–)	superficial gastritis/atrophic gastritis	197/233	117/122	6/13	123/135
		OR (95% CI)	1.000	0.882(0.642–1.21)	1.832 (0.684–4.909)	0.937(0.687–1.279)
	(+)	superficial gastritis/atrophic gastritis	107/109	74/70	4/2	78/72
		OR (95% CI)	0.861(0.621–1.195)	0.8(0.548–1.167)	0.423(0.077–2.333)	0.782(0.538–1.136)
				*P* = 0.621	*P* = 0.308	*P* = 0.566
**Alcohol**	(–)	superficial gastritis/atrophic gastritis	231/263	132/142	9/12	141/154
		OR (95% CI)	1.000	0.945(0.703–1.27)	1.171(0.485–2.829)	0.959(0.719–1.281)
	(+)	superficial gastritis/atrophic gastritis	69/77	52/49	1/3	53/52
		OR (95% CI)	0.98(0.677–1.419)	0.828(0.539–1.27)	2.635(0.272–25.507)	0.862(0.565–1.313)
				*P* = 0.852	*P* = 0.815	*P* = 0.920

*P* values were adjusted for age and sex.

**Table 5:** Interaction between *GSTP1* Ile/Val polymorphism and *H. pylori* infection, smoking, and alcohol consumption in gastric cancer.

			superficial gastritis *vs* gastric cancer			
			Ile/Ile	Ile/Val	Val/Val	Ile/Val + Val/Val
***H. pylori***	(–)	superficial gastritis/gastric cancer	253/168	153/92	10/19	163/111
		OR (95% CI)	1.000	0.906(0.655–1.252)	2.861(1.298–6.306)	1.026(0.752–1.399)
	(+)	superficial gastritis/gastric cancer	90/163	40/82	4/26	44/108
		OR (95% CI)	2.727(1.975–3.767)	3.087(2.018–4.724)	9.789(3.356–28.555)	3.696(2.475–5.521)
				***P*** ** = 0.000**	***P*** ** = 0.000**	***P*** ** = 0.000**
**Smoking**	(–)	superficial gastritis/gastric cancer	136/69	72/32	5/5	77/37
		OR (95% CI)	1.000	0.876(0.527–1.455)	1.971(0.552–7.04)	0.947(0.582–1.542)
	(+)	superficial gastritis/gastric cancer	100/82	67/47	4/12	71/59
		OR (95% CI)	1.616(1.071–2.439)	1.383(0.862–2.217)	5.913(1.839–19.015)	1.638(1.044–2.571)
				***P*** ** = 0.040**	***P*** ** = 0.003**	***P*** ** = 0.023**
**Alcohol**	(–)	superficial gastritis/gastric cancer	170/74	86/32	8/9	94/41
		OR (95% CI)	1.000	0.855(0.524–1.394)	2.584(0.96–6.96)	1.002(0.634–1.583)
	(+)	superficial gastritis/gastric cancer	62/49	48/28	1/7	49/35
		OR (95% CI)	1.816(1.142–2.886)	1.34 (0.781–2.3)	16.081(1.944–133.038)	1.641(0.983–2.739)
				***P*** ** = 0.028**	***P*** ** = 0.000**	***P*** ** = 0.027**

*P* values were adjusted for age and sex.

## Discussion

Over the past 20 years, there has been marked progress in our understanding of the role of genetic and environmental factors in the etiology of gastric cancer. GSTs are multifunctional and multigene products. They are versatile enzymes and participate in the nucleophilic attack of the sulfur atom of glutathione on the electrophilic centers of various endogenous and xenobiotic compounds. Out of the major classes of GSTs, GSTP1 has significance in the diagnosis of cancer because it is expressed abundantly in tumor cells [29]–[31]. It is a single gene product, coded by seven exons. Some studies have observed that a relation between the *GSTP1* polymorphism and GSTP1 is involved in some cellular functions. The best characterized of these is its role as a phase II enzyme in which it catalyzes the S-conjugation of glutathione (GSH) with a wide variety of electrophilic compounds, including many mutagens, carcinogens, anticancer agents, and their metabolites. Polymorphisms of *GSTP1* have been reported, isoleucine (Ile) 105 valine (Val) in exon 5 and alanine (Ala) 114 valine (Val) in exon 6 [32]. The activity of this enzyme is affected by substitution at position 105, which is located in the hydrophobic substrate binding site, and this has considerable affects depending on the type of chemical reaction. It has been suggested that compared with *GSTP1* Ile 105, *GSTP1* Val 105 has a higher catalytic efficiency with regard to the metabolism of carcinogenic aromatic epoxides [33], [34].

In the present case–control study, we reported that the polymorphism of *GSTP1* was significantly associated with an increased risk of gastric cancer in the Chinese population. At present, there are few reports about the association between the polymorphisms of *GSTP1* and the risk of gastric cancer. Researchers in the USA [35] have reported that the *GSTP1* genotype seemed not to be associated with the risk of gastric cancer and chronic gastritis in a high-risk Chinese population. The results detected by Katoh et al [36] suggest the frequency of the *GSTP1* allele Val is increasing in gastric cancer in the Japanese population, but this has not yet obtained statistical significance. We found that there was a significant difference in the *GSTP1* polymorphic types between the gastric cancer cases and superficial gastritis controls. The frequency of *GSTP1* Val/Val genotypes was significantly higher in the gastric cancer group, compared with Ile/Ile or Ile/Val genotypes. The analysis showed a statistically significant 3.324-fold increase in gastric cancer risk associated with the *GSTP1* allele Val. This suggests that individuals from Northern China with *GSTP1* allele Val have an increased risk of gastric cancer, but not atrophic gastritis (one of the precancerous conditions). However, it’s worth mentioning that in subgroups aged >60 years, an increased atrophic gastritis risk associated with Ile/Val genotypes was more evident. These findings revealed that the *GSTP1* Ile/Val polymorphism could affect the stage of gastric carcinogenesis, the extent of atrophic gastritis as a precancerous lesion.

Compared with other studies, we found that the allele Val frequencies of *GSTP1* (21.1%) were significantly different from those in western people, such as European–Americans (33%) and African–Americans (42%) [25], [37]. This suggests the possibility of *GSTP1*-genotype-associated ethnic differences. The fact that, in our study, genotype frequencies among the population fitted the Hardy–Weinberg law further supports this view. The mechanism of the association between *GSTP1* gene polymorphism and gastric cancer was not clear in our study. However, it can be supposed that GSTP1 is a major GST isoform expressed in human gastrointestinal epithelium, which can eliminate DNA oxidative products of thymidine or uracil propenal. After induction by cytochrome P450, some tumor-related carcinogens, such as benzo[a]pyrene diol epoxide and acrolein, can also be eliminated by *GSTP1*. The Ile→Val substitution may be associated with a higher level of DNA adducts, thus increasing the susceptibility to gastric cancer induction.

Furthermore, stratified analyses revealed that subgroups of smoking or alcohol consumption were more likely to have been diagnosed with gastric cancer. Our analysis supports there being an elevated risk of gastric cancer among individuals with *H. pylori* infection, smoking, or alcohol consumption, and the *GSTP1* Val/Val genotype.

*H. pylori* has been assigned as a classs I carcinogen by the World Health Organization, and acts as the initiating agent [38]. Serological *H. pylori* IgG testing was a useful noninvasive strategy for testing for *H. pylori*. It was particularly useful in areas where the prevalence of *H. pylori* was high. We investigated the interaction between *GSTP1* genotype and *H. pylori* infection in gastric cancer and its precancerous conditions. Association of the *GSTP1* Val/Val genotype with *H. pylori* infection significantly increased gastric cancer and atrophic gastritis risk. This important finding suggests that *GSTP1* genotyping and *H. pylori* IgG seropositivity could be used to identify individuals with a high risk of gastric cancer and its precancerous conditions. The mechanism of action might be that the *H. pylori* infection resulting in gastric cancer and its precancerous conditions depends on genetic polymorphisms influencing the virulence of the organism. *GSTP1* Ile/Ile has a higher catalytic efficiency than *GSTP1* Ile/Val or Val/Val for most environmental carcinogens, including cytokines produced by *H. pylori* infection. These cytokines that could not be detoxified by GSTP1 could directly induce gastric mucosal damage and eventually lead to development of atrophic gastritis and even gastric cancer. The exact molecular biology mechanisms need further exploration.

Tobacco smoking and alcohol consumption are the main known etiological factors of some cancers. In this study, we observed that higher ratios of people in the gastric cancer group had consumed tobacco and alcohol (57.1% and 42.2%, respectively), compared with the controls (44.5% and 29.6%, respectively) (Table 1). This finding indicated that alcohol and tobacco consumption are highly associated with increased risk for gastric cancer. Long-term tobacco smoking and alcohol consumption have been shown to contribute to carcinogenesis [39]. Tobacco consumption can significantly increase nuclear hypoxia-inducible factor (HIF)-1a expression, and alcohol can increase protein levels of c-fos and c-jun proto-oncogenes [40], [41]. Association of the *GSTP1* Val/Val genotype with smoking or alcohol consumption could significantly increase atrophic gastritis and gastric cancer risk. This phenomenon might be caused by alterations in catalytic efficiency between tobacco and alcohol constituents and the polymorphic *GSTP1* gene. These findings provide a possible molecular explanation for the synergistic effect of smoking and alcohol consumption on gastric cancer development. However, details of the mechanism must be verified by other well-designed experiments.

In conclusion, our results suggest that polymorphism of *GSTP1* may contribute to gastric cancer susceptibility in the Chinese population. Moreover, the combined effect of *GSTP1* Val allele with environmental carcinogens (*H. pylori* infection, smoking, and alcohol consumption) significantly increases the risk of gastric cancer development.
